# Cost-Utility Analysis of Antibiotic Therapy versus Appendicectomy for Acute Uncomplicated Appendicitis

**DOI:** 10.3390/ijerph18168473

**Published:** 2021-08-11

**Authors:** Ayesha Ali, Zina Mobarak, Mariam Al-Jumaily, Mehreen Anwar, Zaeem Moti, Nadia Zaman, Amir Reza Akbari, Laure de Preux

**Affiliations:** 1 Faculty of Medicine, Imperial College London, London SW7 2AZ, UK; tahmeena.zaman17@imperial.ac.uk; 2 Business School, Imperial College London, London SW7 2AZ, UK; zina.mobarak20@imperial.ac.uk (Z.M.); mariam.al-jumaily20@imperial.ac.uk (M.A.-J.); mehreen.anwar20@imperial.ac.uk (M.A.); zaeem-zunaid.moti20@imperial.ac.uk (Z.M.); 3 Faculty of Biology, Medicine and Health, University of Manchester, Manchester M13 9PL, UK; amir.akbari@student.manchester.ac.uk; 4 Department of Economics and Public Policy, Business School, Imperial College London, London SW7 2AZ, UK; l.depreux@imperial.ac.uk

**Keywords:** economic evaluation, cost-utility, acute uncomplicated appendicitis, appendicectomy, antibiotic therapy

## Abstract

Background: Current UK National Health Service (NHS) guidelines recommend appendicectomy as gold standard treatment for acute uncomplicated appendicitis. However, an alternative non-surgical management involves administrating antibiotic-only therapy with significantly lower costs. Therefore, a UK-based cost-utility analysis (CUA) was performed to compare appendicectomy with an antibiotic-only treatment from an NHS perspective. Methods: This economic evaluation modelled health-outcome data using the ACTUAA (2021) prospective multicentre trial. The non-randomised control trial followed 318 patients given either antibiotic therapy or appendicectomy, with quality of life (QOL) assessed using the SF-12 questionnaires administered 1-year post-treatment. A CUA was conducted over a 1-year time horizon, measuring benefits in quality adjusted life years (QALYs) and costs in pound sterling using a propensity score-matched approach to control for selection based on observable factors. Results: The CUA produced an incremental cost-effectiveness ratio (ICER) of −GBP 23,278.51 (−EUR 27,227.80) per QALY. Therefore, for each QALY gained using antibiotic-only treatment instead of appendicectomy, an extra GBP 23,278.51 was saved. Additionally, two sensitivity analyses were conducted to account for post-operative or post-treatment complications. The antibiotic-only option remained dominant in both scenarios. Conclusion: While the results do not rely on a randomized sample, the analysis based on a 1-year follow-up suggested that antibiotics were largely more cost-effective than appendicectomy and led to improved QOL outcomes for patients. The ICER value of −GBP 23,278.51 demonstrates that the NHS must give further consideration to the current gold standard treatment in acute uncomplicated appendicitis.

## 1. Introduction

### 1.1. Background

The appendix is a small, narrow tube approximately 5 to 10 cm long that is connected to the caecum—the beginning of the large intestine [1]. Acute appendicitis occurs when there is an inflammation of the appendix, causing a painful swelling in the lower right abdomen [1,2]. Unlike complicated appendicitis, uncomplicated appendicitis indicates no evidence of an abscess, a ruptured appendix, or inflammation of the abdominal wall lining [3].

According to the National Institute for Health and Care Excellence (NICE) guidelines, the gold standard treatment for acute uncomplicated appendicitis in the UK is appendicectomy, which involves the surgical removal of the appendix [4]. Around 50,000 appendicectomies are performed annually in the UK, and it is the ‘most common abdominal surgical emergency in the world’ [1]. Laparoscopic appendicectomy (‘keyhole surgery’) is preferred to an open approach as it is associated with less pain, lower incidence of surgical site infections (SSI), decreased length of hospital stay, earlier return to work, and better quality of life scores [1,5]. However open appendicectomy is still used if the appendix has burst or if access is difficult [2].

A newer alternative to appendicectomy, also recommended by NICE, is treating acute uncomplicated appendicitis using a conservative, non-operative approach [1]. Appendicectomy is recommended for all patients with complicated appendicitis and cannot be ruled out [6]. However, many recent studies have shown that intravenous fluids and antibiotic therapy can be an effective alternative in treating uncomplicated appendicitis [5,6,7].

### 1.2. Motivation and Rationale

Appendicitis is one of the most common causes of acute abdominal pain in children and adults [5]. Non-operative therapy for uncomplicated appendicitis is currently of great interest in the surgical community, and there has been an abundance of studies looking into its effectiveness as an alternative treatment approach. Whilst non-operative management can be successful, a recent meta-analysis found that 26.5% of patients on antibiotic therapy needed an appendicectomy within 1 year, resulting in 23.8% lower treatment effectiveness than the surgical group [8]. However, antibiotic-only treatment also achieves a lower overall complication rate at 5 years and shorter sick leave compared to surgery [9]. The use of antibiotics for uncomplicated appendicitis has been limited due to the conflicting evidence and lack of definitive research regarding the efficacy of antibiotics, quality of life, and overall cost-effectiveness compared to surgery. Therefore, there is a clear justification for a UK-based cost-utility analysis (CUA).

### 1.3. Study Objectives

The aim of this study was to carry out a CUA comparing appendicectomy to an antibiotic-only treatment approach for the treatment of uncomplicated appendicitis in adults. Using the available literature, this study will explore the costs and effectiveness of each method to determine which is the most efficient first-line treatment that is able to support the National Health Service’s (NHS) limited healthcare resources.

### 1.4. Literature Review

A search of the literature was conducted on three databases including Embase, Medline, and Google Scholar using the key terms ‘antibiotics’, ‘appendectomy’, ‘appendicectomy’ and ‘appendicitis’. Exclusion and inclusion criteria are reported in Appendix A Table A1. Due to the volume of research regarding this topic as well as advancements in the surgical treatments and antibiotics that are offered, we chose to limit our analysis to studies published in the past five years.

Conservative therapy for uncomplicated appendicitis has garnered interest in recent years, particularly after a randomised control trial in Finland, which was the first to investigate the long-term implications of using antibiotic therapy over appendicectomy for uncomplicated appendicitis [9]. The study found that over 5 years, appendicitis recurred in 39.1% of patients who received antibiotic therapy, yet the antibiotic group tended to suffer from 17.9% less complications and required less absence leave from work [9]. However, the study has been questioned because of its use of open appendicectomy surgery compared to the UK gold-standard of laparoscopic surgery, which is associated with better outcomes and lower rates of complications [10]. Furthermore, the study only used CT imaging to diagnose uncomplicated appendicitis, whilst ultrasound imaging is often used in the UK [11].

In response to these criticisms, a US randomised trial was launched that used laparoscopic surgery as the main way to conduct the appendicectomy [12]. Whilst this trial certainly shows itself to be promising with 1552 participants, the study is ongoing and has only published results showing quality-of-life outcomes after 30 days (showing that antibiotics are non-inferior to appendicectomy) [12]. As demonstrated by the APPAC and the recently published ACTUAA trial (2021), we do not believe this time frame is adequate to truly assess the efficacy of both treatments, as complications may not have fully manifested within a month [13]. A similar problem arises with a UK based study, which also shows health outcomes after a month with no further follow up [14]. Furthermore, in the UK study, 56% of patients in the appendicectomy group received open surgery as opposed to laparoscopic surgery, which can be attributed to the fear of the increased risk of COVID-19 infection using laparoscopic surgery [14].

Though economic evaluations have been conducted on this topic, these have been based on the APPAC trial, which used mainly open appendicectomy over laparoscopic surgery and has not been conducted from the NHS perspective [15,16]. These economic evaluations tend to focus more on economic factors as opposed to quality-of-life outcomes when comparing the two treatments. Sceats et al. (2019) assess health outcomes and costs in their analysis; however, the data is based purely from an American perspective [17]. To our knowledge, this will be the first study conducting a cost-utility analysis of antibiotic therapy versus appendicectomy for uncomplicated appendicitis using data from the ACTUAA trial and UK costing.

## 2. Methods

Given the notable limitations with the studies identified in the literature review, our analysis was therefore modelled based on outcomes from the Italian ACTUAA trial, which was conducted from 2017 to 2018 with the last follow-up in 2019 [13]. The non-randomised control trial followed 318 patients given either antibiotic therapy or appendicectomy, with quality of life assessed using SF-12 questionnaires both after 1 month and 1 year following treatment. Patients ranged from 18 to 65 years.

### 2.1. Propensity Score Matching

To minimise selection bias from the non-randomised nature of this trial, a propensity score analysis was conducted to match participants. Propensity score matching is often used in cases where two populations have large differences in their covariates, and these differences can lead to biased outcomes [18]. Covariates are balanced in propensity score matching in order to reduce this bias [18].

The authors matched 87 participants from the antibiotic treatment group with 87 participants from the appendectomy treatment group. Propensity scores were calculated based on age, sex, AIR score on admission, WBC count, % of neutrophils on admission, previous episodes of acute appendicitis, time from symptom onset to treatment, and antibiotic therapy prescription. Participants were matched using ‘nearest neighbour matching’ based on their individual propensity scores with a caliper set at 0.2 and a 1:1 matching model with replacement. This allowed the authors to ensure that the two treatment groups were balanced and mitigated the effects of confounding variables on the quality-of-life outcomes post-treatment.

The authors referred to these 174 patients as the ‘post-matching’ sample, with the original 318 patients referred to as the ‘pre-matching’ sample. Therefore, to reduce impact of bias, data from the 174 post-matching sample were utilised in our study. Differences between the pre- and post-matching samples are reported in Appendix B Table A2.

### 2.2. Application to a UK Based Health System

Though this trial was conducted in Italy, the results of the study were applied to the healthcare system in the United Kingdom and considering the similarities between the British and Italian healthcare systems. First, both have national healthcare services as in the UK, there exists the NHS, and in Italy, there is the Servizio Sanitario Nazionale (SSN) [19]. Both the NHS and SSN are financed by their respective governments through income tax and national insurance contributions [19]. The Department of Health and Social Care in the UK and the Ministry of Health in Italy are the government bodies that set health policies and deal with legislation [20]. Furthermore, as organisations, both the NHS and SSN were founded based on key principles. These include the provision of health services that are predominantly free at the point of use, access to healthcare based on need, irrespective of socioeconomic background, and universal coverage [21].

Following the application of the results to a UK-based health system, the costs of the two procedures and their subsequent complications were extracted from NHS National Tariffs, NHS Reference Costs, the British National Formulary (BNF), and scientific papers.

### 2.3. Choice of Analysis

A cost-utility analysis was chosen for this study. Monetary units (GBPs) were used for the cost of antibiotic treatment, appendicectomy, and complications that could arise. The obtained results are also reported in Euros, utilising an exchange rate of EUR 1.1697 as of 2 August 2021 [22]. A CUA was conducted over a cost-benefit analysis (CBA) or a cost-effectiveness analysis (CEA), as health outcomes are measured in Quality Adjusted Life Years (QALYs) [23]. This is a standardised measure that takes into consideration the length of life and the quality-of-life post-intervention and allows for comparison between the two treatments [23,24]. Furthermore, NICE uses QALYs in their decision-making process on resource allocation, making QALYs the optimal unit for a UK-based CUA [25].

### 2.4. Choice of Perspective

The analysis was conducted from the NHS perspective. Due to its limited budget and opportunity cost considerations when providing healthcare, the need to find cost-efficient treatment options that also maximise health outcomes for patients remains of vital importance [25]. As the NHS provides treatment free of charge, we did not consider the patient perspective in our analysis.

### 2.5. Time Period

Our study is modelled on the ACTUAA trial, which followed patients over a one-year period. Although the study also reported outcomes after a month following treatment, these were vastly different to the yearly outcomes, so we determined that one month was not an adequate time frame to measure complications such as appendicitis recurrence. The APPAC study (2018) was the first to explore outcomes over a longer period of time; however, 70% of complications occurred within the first year of treatment [9]. In their meta-analysis, Yang et al. (2019) recommend that efficacy should be judged over a year, as this was the standard commonly used by the literature to judge complications post-antibiotic treatment [26]. 

### 2.6. Modelling

**Figure 1:**
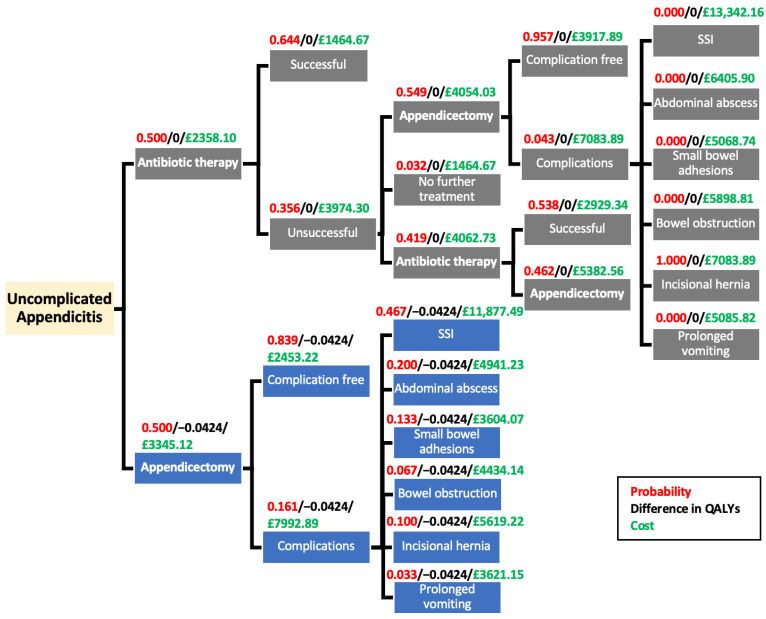
Decision tree.

**Figure 2:**
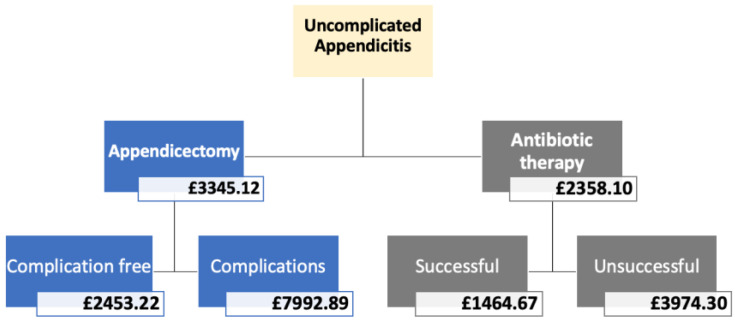
Costs extracted from the decision tree.

A decision tree was modelled using the outcomes reported in the ACTUAA trial over a period of a year and is presented in Figure 1 below [13]. Cost data was obtained from Appendix C Table A3 and Table A4 and is presented in a simplified diagram in Figure 2.

Figures used in the tree were those reported from the post-matching sample to minimise bias from the non-randomised nature of the study. A limitation of this method meant that a smaller sample size of 174 patients was included in the analysis as opposed to the original 318 patients enrolled in the trial.

For the appendicectomy arm of the tree, the treatment was deemed successful if no post-complications occurred (*n* = 73). The complications included in the tree were those that were reported in the study and also echoed those that are reported by NICE for appendicectomy procedures [4]. It was assumed that the probability for each type of complication occurring was the same for the pre- and post-matching appendicectomy groups, as complications were only reported for the pre-matching appendicectomy group.

Although death is a consideration when conducting surgical procedures, no patient deaths were reported in the study, so they were not investigated as an outcome in the tree. Furthermore, the NHS reports that serious complications from laparoscopic surgery are rare and can usually be corrected using further treatment options [27].

For the antibiotic therapy arm, the treatment was deemed unsuccessful (*n* = 31) if the patient experienced a recurrence of appendicitis following discharge or if the treatment failed to heal the appendicitis in the first place. Patients who experienced treatment failure were then assigned to experience a further cycle of antibiotics (*n* = 13) or were referred to have an appendicectomy (*n* = 17). One patient did not receive either treatment, so it was assumed that they received no further interventions. Those who showed no success with further cycles of antibiotic therapy were then referred to have an appendicectomy (*n* = 6). Of these patients, the study reports no post-operative complications, which were therefore not recorded in the tree.

Notably in the study, the probability of complications occurring for the appendicectomy group after surgery was much higher than for those who underwent an appendicectomy after antibiotic therapy failure. These differences will be explored more thoroughly in the sensitivity analysis.

### 2.7. Costs

#### 2.7.1. Discount Rate

As the ACTUAA trial did not report monetary costs for the intervention; these were obtained from sources that gave costs in pounds (GBP). Costs obtained from sources originating before 2021 were inflated by an annual rate of 3.5%, in line with NICE guidelines [25]. All costs included in the decision tree reflect 2021 values and are referenced in Appendix C Table A3 and Table A4.

#### 2.7.2. Appendicectomy

The cost of an appendicectomy procedure was taken from Clement et al. (2020), who investigated costs for laparoscopic surgery in the NHS [28]. The total appendicectomy cost included factors such as length of stay, cost of imaging, and medications. Though 24.1% of patients in the ACTUAA study underwent open surgery, the authors reported no difference in outcomes or complications between the two methods of surgery. Therefore, for the purpose of our analysis, the two were grouped under ‘appendicectomy’ and using laparoscopic surgery costs, as this was the dominant method conducted.

The study reports that the mean hospital stay for patients in the appendectomy group was 3.4 days ± 1.5 SD (excluding patients diagnosed with complicated appendicitis who had longer stays). It was assumed that patients who developed post-operative complications stayed in hospital for longer than those who had complication-free treatment. Using the ‘range rule of thumb’ which states that the standard deviation of a sample is a quarter of the range of a sample, the number of days patients with complications stayed in hospital was calculated to be 3.4 + 2 (1.5) equaling 6.4 days [29]. Therefore, the extra costs for those with complications were calculated over 3 days (6.4 − 3.4 = 3 extra days in hospital), using NHS tariff values for cost per daily hospital stay [30].

Treatment doses for complications were assumed to be given over the 3 days. The costs of treatment doses were then added on to the costs per extra day spent in hospital to calculate complications post-appendicectomy. The costs of the medications were obtained from the British National Formulary (BNF), and procedure costs were obtained from the NHS’s 2019/20 National Tariff Payment System [31].

#### 2.7.3. Antibiotic Therapy

The total cost for one cycle of antibiotic therapy was calculated by adding the cost of hospital stay, imaging, blood tests, and antibiotics together. The cost of hospital stay was calculated by the mean length of hospital stay reported in the trial for the antibiotic group (3.1 days after complicated appendicitis was excluded) multiplied by the cost per day of hospital stay. This cost was obtained and inflated from the NHS Reference Costs 2017/18 [30].

There were six different antibiotic regimens that were used in the trial [13]. The cost of each antibiotic was obtained from the BNF, as this gives updated and reliable data on drug costs [32,33,34,35,36,37,38]. This gave the cost per unit, which was then multiplied by the dosage and mean length of hospital stay reported in the trial. A weighted average was then worked out using the number of patients that underwent each regimen in the trial.

The cost of blood tests and imaging were obtained and inflated from the Clement et al. (2020) study in order to ensure that these values remained consistent across the two treatment groups [28].

### 2.8. Benefits

The ACTUAA trial (2021) assessed participant QOL via the SF-12 score at 1 year. The health outcomes for this CUA were measured as a difference in QALYs, where one QALY means one year of perfect health [23]. This is calculated by multiplying the utility value by years lived in that state.

QALY = Quality of Life (QOL) × Length of Life (LOL)

The SF-12 score uses a scale of 0 to 100, which was re-scaled to 0 to 1 to calculate the QALYs. This value was then multiplied by 1 year, which was the length of follow up for the trial. Although the study was conducted from 2017 to 2018, QALY values were not inflated, as it was assumed that the QALY values obtained would remain constant across time.

The SF-12 score reported in the trial was an overall difference between the antibiotic treatment group and the surgical group, with the antibiotic group being reported as a reference. Therefore, instead of using individual QALY values, only the difference in QALY could be used as reported in Table 1. This difference represented the end-value for the treatment options, taking into account all of the complications or health outcomes that would have occurred from each. This meant the QALY values were kept constant throughout each arm of the decision tree. The antibiotic treatment group was assigned a value of 0, meaning no difference in QALY—as this was the reference group—and the appendicectomy group was assigned the difference −0.0424.

## 3. Results

From the decision tree (Figure 1), the expected costs of antibiotic treatment and appendicectomy were found to be GBP 2358.10 (EUR 2758.16) and GBP 3345.11 (EUR 3912.62), respectively. The expected appendicectomy cost is higher, primarily due to the operative and complications cost, as there was only a 0.3-day difference in the mean hospital stay. The small difference in costs can be attributed to patients in the antibiotic group having a recurrence and either needing a second cycle of antibiotics or an appendicectomy within a year, increasing the overall cost for the antibiotic group. The Incremental Cost-Effectiveness Ratio (ICER) was calculated by dividing the difference in cost (GBP 2358.19–GBP 3345.11) by the difference in effects (0.0424):

$$ICER=\frac{Cost_{antibiotic}-Cost_{appendicectomy}}{Effects_{antibiotic}-Effects_{appendicectomy}}=\frac{\Delta C}{\Delta E}=\frac{-987.01}{0.0424}=-\mathrm{GBP}\;23,278.51$$

**Figure 3:**
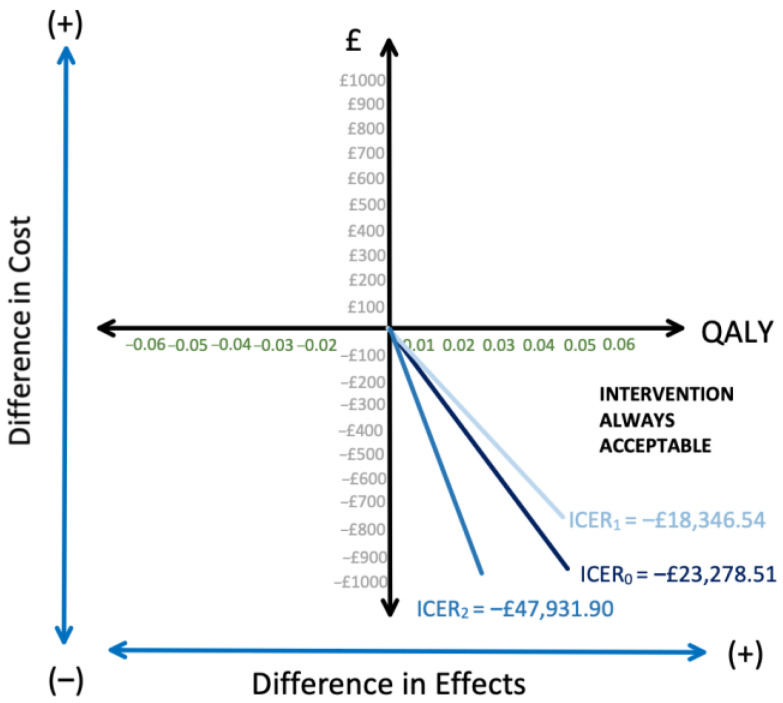
Cost-effectiveness plane diagram; ICER 0 = original; ICER 1 = sensitivity analysis 1; ICER 2 = sensitivity analysis 2.

An ICER Of −GBP 23,278.51 (−EUR 27,227.80) was calculated. This means that for each QALY gained using antibiotic treatment, an extra GBP 23,278.51 (EUR 27,227.80) is saved. NICE uses an ICER threshold of GBP 20,000–GBP 30,000 (EUR 23,393.08–EUR 35,089.62) as reference to determine if a new treatment method is cost-effective for the NHS [39]. The negative ICER is far below the threshold, as the expected benefit of the antibiotic treatment is larger than the one of the appendicectomy, and at the same time, the expected cost of the antibiotic approach is lower than its comparator. This is illustrated in the cost effectiveness plane diagram in Figure 3. The result falls in the lower right quadrant, implying that it is always superior to its comparator [25].

Using a conservative threshold of GBP 20,000 (EUR 23,393.08) per QALY, the Monetary Net Benefit (MNB) and Health Net Benefit (HNB) were calculated to be greater than 0, supporting the conclusion that antibiotic-first treatment is cost-effective.

$$MNB=\left(R_{c}*\Delta E\right)-\Delta C=\left(\mathrm{GBP}\;20,000*0.0424\right)--987.01=\mathrm{GBP}\;1835.01\;(\mathrm{EUR},\;2146.33)$$

$$HNB=\Delta E-\frac{\Delta C}{R_{c}}=0.0424-\frac{-987.01}{\mathrm{GBP}\;20,000}=0.092$$

### 3.1. Sensitivity Analysis

There were two one-way sensitivity analyses that were performed to ensure the robustness of our conclusions.

#### 3.1.1. Sensitivity Analysis 1

Post-operative outcomes in the ACTUAA trial differed between the appendicectomy treatment group and those who received an appendicectomy after antibiotic treatment failure. The rate of complications for the latter group was lower than that of the former group, leading to decreased costs and a different ICER value. Such differences may be due to the fact that the sample size for those who experienced an appendicectomy after antibiotic therapy was much lower (*n* = 17 for the ‘antibiotic + appendicectomy’ group and *n* = 6 for ‘antibiotics + antibiotics + appendicectomy’ group) compared to the appendicectomy group (*n* = 231 pre-matching). Consequently, complications may have occurred at a lower rate and would not have been truly representative [40].

Therefore, the rate of complications from the appendicectomy only group, which had a bigger sample size, was applied to the groups that had an appendicectomy after antibiotic therapy, and the costs were recalculated for the antibiotic groups. Despite the increased costs for the antibiotics group, the ICER value obtained was −GBP 18,346.54 (−EUR 21,457.85) which remained favourable for antibiotics (Figure 3).

#### 3.1.2. Sensitivity Analysis 2

In the ACTUAA study, the mean SF-12 quality of life scores was given for both treatment groups. Consequently, those who suffered from complications from either treatment group could not be distinguished from those who had successful treatment when assigning QALYS. Post-operative complications, such as SSI, can certainly have an impact on quality of life, as Guest et al.’s (2018) study shows that the incurred wound can persist for months and requires thorough attention and additional healthcare visits [41]. Therefore, for this sensitivity analysis, we utilised the 95% confidence intervals reported in the study for the mean SF-12 scores to assign the revised QOL differences.

Confidence intervals report the range of plausible values for the mean, and for the mean difference in QOL, this was given to be −4.24 (−6.34, −2.15) [42]. Hence, for this analysis, a difference of −6.34 reflected the largest difference in the mean QOL scores from the reference group (antibiotic therapy), and −2.15 was the smallest difference. Successful antibiotic therapy was kept as the reference group at 0; however, those who suffered from complications in the appendicectomy group were assigned the largest difference of −6.34, assuming that they suffered the largest decrease in QOL. Both complication-free appendicectomy and complications with antibiotics were assigned the minimum difference of −2.15 (Table 2).

A new ICER value of −GBP 47,931.90 (−EUR 56,063.74) was obtained, again remaining favourable for antibiotic therapy.

## 4. Discussion

In the ACTUAA trial, despite the superior SF-12 score and antibiotics being deemed a safe option, the study still concluded that appendicectomy ‘undoubtedly remains the most effective treatment for patients with acute appendicitis’ [13]. This conclusion was reached without a systematic economic evaluation of both options, and the conclusion was relying only on the statistically significantly higher complication-free treatment success of the appendicectomy. However, Sippola et al.’s (2020) study showed that out of the 81 patients in the APPAC antibiotic-first group who underwent an appendicectomy, 33% would still choose antibiotics as their primary treatment [43]. Undeniably, there is much debate about which treatment method is superior, and more research is needed with larger and randomised samples. However, the results of this cost-utility analysis demonstrate that an antibiotic-first treatment approach has better health outcomes and is less costly for the NHS than an appendicectomy. Therefore, NICE should consider its implementation as a first-line treatment for acute uncomplicated appendicitis.

### 4.1. Comparison of the Results with Literature

There have been no cost-utility analyses of antibiotic therapy versus laparoscopic appendicectomy for uncomplicated appendicitis from a UK perspective. Upon comparing our results to Sippola et al.’s (2017) and Haijanen et al.’s (2019) studies, there is a common conclusion that antibiotic therapy did in fact result in lower costs than surgery [15,16]. In contradiction to this, Sceats et al.’s (2019) study—which used QALYs to generate an ICER —found that laparoscopic appendicectomy was more cost effective and resulted in better health outcomes [17]. However, this study was conducted using a US-based healthcare setting and costing, which differs significantly from that of the UK.

### 4.2. Limitations

The findings of this economic evaluation should be considered in the context of several limitations. Appendix D Table A5 highlights the key assumptions made in the analysis.

Although every effort was made to ensure the costs of treatment for the interventions and subsequent complications were collected from the same source, this was not always possible, as the NHS tariff payment system did not supply all of the cost data needed to construct the decision tree. Therefore, various sources were used to collect cost data for our study, including the BNF, NHS National Tariffs, NHS Reference Costs, and scientific studies. We recognise that this variation may have biased the final costs that were calculated for each intervention.

In the ACTUAA trial, patients who were diagnosed with complicated appendicitis at the imaging stage were excluded from the study. However, it is important to note that 20 patients of the 174 included in the analysis went on to develop complicated appendicitis later on and may have been more prone to operative complications and antibiotic treatment failure [5]. These patients could not be excluded from the decision tree, as their outcomes were also included in treatment success and failure rates of the two interventions.

Furthermore, the study reported quality of life scores as a difference between the two treatments as opposed to providing separate figures for each treatment with its complications. Therefore, our decision tree could only provide the difference in the SF-12 scores and remained the same for each branch despite complications that may have impacted quality of life.

Finally, it is important to consider that the ACTUAA trial was a non-randomised control trial, and though bias was minimised using post-matching samples, we cannot exclude that patients expected to have better health outcomes with antibiotic treatment may have been selected for this group.

### 4.3. Generalisability

The ACTUAA trial was conducted in Italy, and due to the similarities between the healthcare systems in the two countries, the outcomes were generalised to the UK population. Both the UK and Italy share similar population characteristics as well as possess a national health care system with free-of-charge coverage [44]. However, there may be differences in medical procedures and antibiotic regimens that affect outcomes. As the sample was not randomised, matching had to be implemented, which resulted in a relatively small sample size of 174 patients (in the post-matching sample), which was possibly not fully representative of the UK. Furthermore, the costs reported in this study were sourced from NHS-based data and therefore are specific to a UK perspective. This limits the generalisability of this CUA to the wider global context, where healthcare systems vary massively.

## 5. Conclusions

The management of uncomplicated appendicitis is starting to see a shift from the traditional ‘one-size fits all’ operative approach to a consideration of antibiotics as an alternative treatment. The results of this CUA, that antibiotics are more cost-effective than laparoscopic appendicectomy, call for a further consideration by NICE as to what the recommended first-line treatment should be for uncomplicated appendicitis when taking into account both the limited NHS resources and optimal patient outcomes.

## Figures and Tables

**Table 1:** QALY scores.

	SF-12 Score from Trial	Assigned Difference in QALY
Antibiotic-first	Reference	0
Appendicectomy	−4.24	−0.0424

**Table 2:** Sensitivity analysis 2.

Group	Original Difference in QALY	Assigned Difference in QALY
Antibiotics-Successful	0 (reference group)	0 (reference group)
Antibiotics-Unsuccessful	0 (reference group)	−0.0215
Appendicectomy-Complications	−0.0424	−0.0634
Appendicectomy-No complications	−0.0424	−0.0215

## Data Availability

All data used are referenced and are available within the manuscript and the Appendix.

## Appendix A

**Table A1:** Criteria for literature review.

Inclusion	Exclusion
Studies published in and after 2016	Paediatric appendicitis
Randomised and non-randomised control trials	Non-English studies
Cohort studies	Studies investigating complicated appendicitis
Economic evaluations	Ideas, editorials, or opinions

## Appendix B

**Table A2:** Pre- and post-matching samples used for propensity score-matched analysis in the ACTUAA trial [13].

Comparisons	Pre-Matching			Post-Matching		
	Antibiotic	Appendicectomy	*p* Value	Antibiotic	Appendicectomy	*p* Value
Patients: *n* (%)	87 (27.4)	231 (72.6)	-	87 (50)	87 (50)	-
Age: mean ± SD	38.4 ± 18.4	40.0 ± 18.0	0.509	38.4 ± 18.4	38.4 ± 18.4	0.990
Sex M:F (M%:F%)	38:49 (43.7 vs. 56.3)	134:97 (58.0 vs. 42.0)	0.024	38:49 (43.7 vs. 56.3)	38:49 (43.7 vs. 56.3)	0.990
Appendicitis inflammatory response (AIR) score: mean ± SD	5.7 ± 2.0	6.9 ± 2.1	<0.00001	5.7 ± 2.0	6.9 ± 2.2	0.00016
Length of primary hospital stay (days): mean ± SD	3.1 ± 0.9	3.4 ± 1.9	0.158	3.1 ± 0.9	3.4 ± 1.5	0.111
SF-12 score at 1-year follow-up (β (95% CI))	Reference	−2.67 (−4.95, −0.38)	0.02	Reference	−4.24 (−6.34, −2.15)	<0.01

## Appendix C

**Table A3:** Costs of Antibiotic therapy (the costs for mean days in hospital, blood tests, ultrasound, and CT were added to the weighted average of antibiotics to calculate the total).

Description	Year	Unadjusted Cost	Inflated (3.5% pa)	Reference	Comment
Mean Days in hospital = 3.1 days	2018	GBP 346 × 3.1 = GBP 1072.60	GBP 1189.21	ACTUAA 2021; [13] NHS 2017/18 Tariffs [30]	
Blood tests	2019	GBP 39.18	GBP 41.97	Clement et al. 2020 [28]	
Ultrasound	2019	GBP 52	GBP 55.70	Clement et al. 2020 [28]	
CT abdo/pelvis	2019	GBP 101	GBP 108.19	Clement et al. 2020 [28]	
Piperacillin-tazobactam 4.5 g every 8 h	2021	GBP 113.82	GBP 113.83	BNF 2021 [32]	Price per day = GBP 36.72 GBP 36.72 × 3.1 = GBP 113.82
Ceftriaxone and metronidazole 1–2 g every 12 h-ceftriaxone 500 mg every 8 h-metronidazole	2021	GBP 88.20	GBP 88.20	BNF 2021 [33,34]	Price per day = GBP 28.45 GBP 28.45 × 3.1 = GBP 88.20
Ceftazidime 1–2 g every 8 h	2021	GBP 26.35	GBP 26.35	BNF 2021 [35]	Price per day = GBP 8.50 GBP 8.50 × 3.1 = GBP 26.35
Amoxicillin-clavulanic acid (co-amoxiclav) 1–2 g every 8 h	2021	GBP 32.86	GBP 32.86	BNF 2021 [36]	Price per day = GBP 10.60 GBP 10.60 × 3.1 = GBP 32.86
Ciprofloxacin and metronidazole 400 mg every 12 h-ciprofloxacin 500 mg every 8 h-metronidazole	2021	GBP 136.56	GBP 136.56	BNF 2021 [37]	Price per day = GBP 44.05 GBP 44.05 × 3.1 = GBP 136.56
Ertapenem 1 g every 24 h	2021	GBP 98.12	GBP 98.12	BNF 2021 [38]	Price per day = GBP 31.65 GBP 31.65 × 3.1 = GBP 98.12
Weighted average calculation	[(113.83 × 27.6) + (88.20 × 11.5) + (26.35 × 16.1) + (32.86 × 35.6) + (136.56 × 8) + (98.12 × 1.2)]/100 = GBP 69.60				
Antibiotics Total	GBP 1464.67				

**Table A4:** Costs of appendicectomy (the cost for extra days in hospital was added on to each complication to produce a total cost).

Description	Year	Unadjusted Cost	Inflated (3.5% pa)	Reference	Comment
**Appendicectomy Total**	2019	GBP 2290.11	**GBP 2453.22**	Clement et al. 2020 [28]	Costs for adults
**Complications**					
3 days in hospital for complications = (72 h)	2018	GBP 346 × 3 = GBP 1038	GBP 1150.85	ACTUAA 2021; [13] NHS 2017/18 Tariffs [30]	3.4 + 2 (1.5) = 6.4 days using ‘range rule of thumb’ 6.4 − 3.4 days = 3 days
Infected surgical wound	2016	GBP 6966	GBP 8273.42	Guest et al. 2018 [41]	Costs incurred for an infected surgical wound over 12 months
**Surgical site infection (SSI) total**			**GBP 9424.27**		
Percutaneous Drainage	2020	GBP 730	GBP 755.55	NHS 2019/20 Tariffs; [31] BMJ Best Practice 2021 [45]	
Meropenem	2021	GBP 185.65	N/A	BNF 2021 [46]	1 g every 8 h: 9 doses
Metronidazole	2021	GBP 20.12	N/A	BNF 2021 [34]	500 mg every 12 h: 6 doses
Ampicillin	2021	GBP 375.84	N/A	BNF 2021 [47]	2 g every 6 h: 12 doses
**Postoperative abdominal abscess total**			**GBP 2488.01**		
**Small bowel adhesions syndrome total**			**GBP 1150.85**	BMJ Best Practice, 2021 [48]	No treatment recommended
Abdominal Procedure	2020	GBP 802	GBP 830.07	NHS 2019/20 Tariffs; [31] BMJ Best Practice 2021 [49]	Emergency therapeutic general abdominal procedure
**Bowel obstruction total**			**GBP 1980.92**		
Incisional hernia procedure	2020	GBP 1947	GBP 2015.15	NHS 2019/20 Tariffs [31]	
**Incisional hernia total**			**GBP 3166**		
Ondansetron (anti-emetic)	2021	GBP 17.08	N/A	BNF 2021 [50]	4 mg per dose. Minimum 3 doses over 3 days
**Prolonged vomiting**			**GBP 1167.93**		

## Appendix D

**Table A5:** Key assumptions made in the analysis.

(1)	Probability of each post-operative complication occurring was assumed to be the same for the pre- and post-matching appendicectomy group.
(2)	Those with post-operative complications represented the maximum value of days stayed in hospital when calculating the range from standard deviation.
(3)	Treatment for post-operative complications was given over the 3 extra days in hospital.
(4)	Laparoscopic and open surgery experienced similar outcomes in this study.
(5)	Italian and UK populations exhibit similar characteristics when considering uncomplicated appendicitis.
